# The Startψell initiative in Action: A Project supporting Early-career Consultant Psychiatrists in a Southeast UK mental health trust.

**DOI:** 10.1192/j.eurpsy.2024.359

**Published:** 2024-08-27

**Authors:** S. Deepak, M. Ghazirad, M. Lowe

**Affiliations:** ^1^Child & Adolescent Mental Health Services, Berkshire Healthcare NHS Foundation Trust, Reading; ^2^Neuropsychiatry, St. Andrews Healthcare, Northampton; ^3^Liaison Psychiatry, Berkshire Healthcare NHS Foundation Trust, Reading, United Kingdom

## Abstract

**Introduction:**

Transition to a consultant role is a challenging time for an early career psychiatrist, as the high level of structure and guidance available during psychiatric training ceases when training is complete. The Royal College of Psychiatrists, UK therefore pioneered the Startψell initiative to ease this transition. This consultant-led initiative proposed embedding good habits and robust coping mechanisms early on, to enable psychiatrists to thrive in their roles and is based on six main pillars (New consultants (StartWell) (rcpsych.ac.uk) **Image 1: Startwell framework.** Berkshire Healthcare NHS Foundation Trust (BHFT) is a mental healthcare provider based in Southeast England where we designed a project to enhance support to new consultants, based on Startψwell principles.

**Objectives:**

The aims of this project were to:

- Improve the experience of early career psychiatrists taking on their first consultant role in BHFT.
- Ensure that the new consultants are provided with relevant information and resources to fulfil their roles safely.
- Set up a system for ongoing support for new consultants till they complete five years in their post.

**Methods:**

Having discussed the feasibility of setting up a local Startwell initiative in BHFT, plans were presented to the medical director and medical staff committee including new consultants, for their input. Under the guidance from senior consultants, monthly meetings were arranged which offered professional development talks and peer support. As several themes emerged at these meetings, we stratified and aligned these local to the RCPsych Startψell framework **(Image 2:Themes).** An induction folder was also collated with all the relevant information in paper and digital format. In addition, a yearly bespoke induction event for new consultant psychiatrists was delivered from 2019-2022 except in 2021, during the pandemic.

**Results:**

We used Likert scales to gather quantitative feedback **(Table 1)** with free box for comments to capture qualitative feedback **(Image 3).** Feedback response rate for the three cohorts were 75%, 70% and 80% respectively.

**Table:** 

	2019	2020	2022
**Overall Satisfaction**	100%	75%	100%
**How relevant was the content of the programme to your new role?**	70%	100%	100%
**How far did the programme meet your expectations?**	100%	75%	100%

**Image:**

**Figure:**
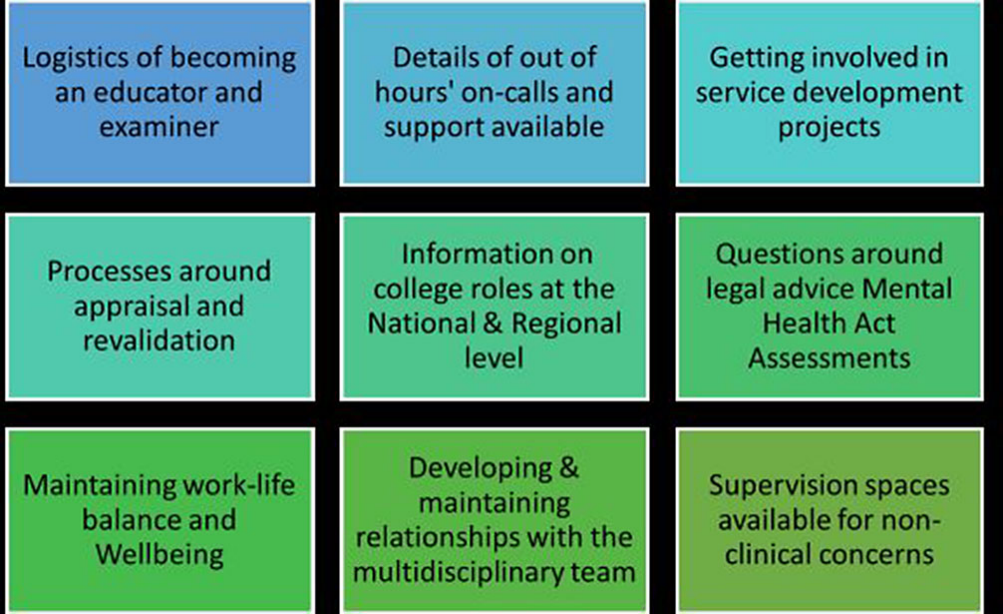

**Image 2:**

**Figure:**
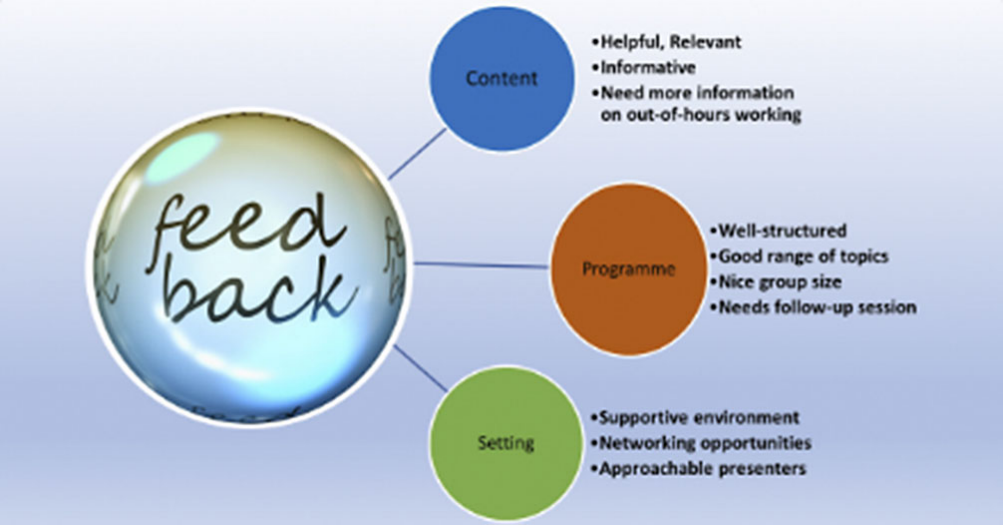

**Image 3:**

**Figure:**
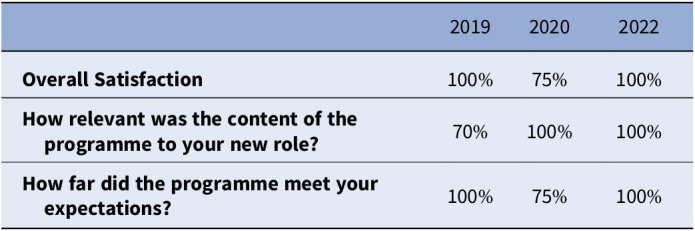

**Conclusions:**

The BHFT Startwell initiative has been running successfully for the past five years and been valued by the new consultants. We are therefore continuing to working towards embedding the programme futher and ensuring sustainably for the future. W e are looking to share our experience in the hope that similar programmes are set up and our newer colleagues can enjoy a long, fulfilling, and enjoyable career.

**Disclosure of Interest:**

None Declared

